# Modified Rush Technique With Plantaris Tendon Augmentation for Chronic Achilles Tendon Rupture: Surgical Technique and Preliminary Clinical Outcomes

**DOI:** 10.7759/cureus.108625

**Published:** 2026-05-11

**Authors:** Nazim Sifi, Ammar Radjai, Abdelkader Metlaine

**Affiliations:** 1 Orthopaedic and Trauma Surgery Unit, Hôpital Pasteur, Colmar, FRA; 2 Orthopaedic and Trauma Surgery Unit, Établissement Public Hospitalier Ouled Djellal, Ouled Djellal, DZA; 3 Orthopaedic and Trauma Surgery Unit, Établissement Public Hospitalier Tenes, Tenes, DZA

**Keywords:** biological repair, chronic achilles rupture, gastrosoleus fascial flap, plantaris tendon, tendon reconstruction

## Abstract

Background

Chronic Achilles tendon rupture remains a challenging condition, particularly in cases involving large tendon defects that preclude end-to-end repair. Among reconstructive options, techniques using local tissues avoid the donor-site morbidity associated with tendon transfers. This study describes a modified gastrosoleus fascial turndown flap based on the Rush technique, reinforced with the plantaris tendon, and reports preliminary clinical outcomes.

Methodology

A total of 13 patients presenting with chronic Achilles tendon rupture (≥4 weeks) with a defect ≥6 cm were included. All underwent reconstruction using a tubularized gastrosoleus fascial flap based on the Rush principle, combined with plantaris tendon augmentation in a framing configuration. Outcomes were assessed using the Achilles Tendon Total Rupture Score (ATRS), the American Orthopaedic Foot & Ankle Society (AOFAS) score, and the ability to perform heel raises. Body mass index (BMI) was analyzed descriptively as an exploratory variable, given the limited sample size precluding formal statistical analysis.

Results

At a mean follow-up of 16.1 ± 2.7 months, the mean ATRS was 81.0 ± 3.9, and the mean AOFAS score was 83.9 ± 2.3. Patients performed a mean of 14.5 ± 1.3 single-leg heel raises. No reruptures or complications were observed. The mean BMI was 25.2 ± 3.4 kg/m², with a slight decrease in ATRS observed in overweight patients.

Conclusions

The modified Rush technique with plantaris tendon augmentation appears to be a reproducible biological option for the management of chronic Achilles tendon ruptures with large defects. In this preliminary series, satisfactory functional outcomes were observed, supporting the feasibility of a fully biological reconstruction strategy using local tissues without tendon sacrifice. This approach may represent a potential alternative to tendon transfers for defects ≥6 cm.

## Introduction

Chronic Achilles tendon rupture is a challenging condition due to tendon retraction, poor tissue quality, and the frequent presence of interposed scar tissue within the defect. When diagnosis is delayed, end-to-end repair is often not feasible, particularly in cases of large defects [1]. Numerous surgical techniques have been described to restore tendon continuity, including V-Y advancement, tendon transfers, particularly flexor hallucis longus (FHL), free grafts, synthetic materials, and local fascial flaps with or without augmentation. Each option has advantages and limitations. Tendon transfers provide dynamic reinforcement but at the cost of donor-site morbidity and potential biomechanical alteration. Free grafts and synthetic materials carry risks of infection or inflammatory reactions. In contrast, local tissue techniques preserve functional structures and offer biologic reconstruction with favorable anatomical and vascular integration [1-3]. Rush described a reconstruction technique based on a gastrosoleus aponeurotic flap harvested adjacent to the defect and tubularized to recreate a tendon-like structure bridging the gap between proximal and distal stumps [4]. This method builds upon Bosworth’s work [5] but differs by creating a broader flap that is converted into a tube, resulting in a structure more closely resembling native tendon morphology. The plantaris tendon has also been used in Achilles tendon reconstruction, primarily as an augmentation structure. It offers several advantages, including anatomical proximity, ease of harvesting, and minimal functional impact, although its role remains largely passive [6,7]. In this context, combining a tubularized fascial flap with plantaris tendon reinforcement appears to be a promising strategy that may provide both biological bridging and additional mechanical support. This study aimed to describe a modified Rush reconstruction technique reinforced with plantaris tendon framing and to report preliminary clinical outcomes in patients presenting with chronic Achilles tendon rupture of ≥4 weeks’ duration and a defect ≥6 cm.

## Materials and methods

Patient population

This retrospective series included 13 patients treated for chronic Achilles tendon rupture by three surgeons across three centers, with a mean follow-up of 16.1 ± 2.7 months. Chronic rupture was defined as a delay of ≥4 weeks. Given the rarity of chronic Achilles tendon ruptures, no specific inclusion or exclusion criteria were applied. All patients who met our definition of chronic rupture and underwent surgical treatment during the study period were included consecutively, without additional selection criteria. Data were collected retrospectively from medical records, operative reports, and follow-up consultations. Collected variables included demographic data (age, sex, body mass index (BMI)), comorbidities (including diabetes), smoking status, activity level, occupational status, injury mechanism, and intraoperative findings such as tendon defect size. All patients presented with functional impairment, characterized by persistent ankle weakness and an inability to ambulate normally. The mean age was 44.6 years (range = 33-68), and all participants were male. No patients had peripheral vascular disease, active infection, or a history of prior ipsilateral surgery. The mean age was 44.6 years (range = 33-68), and all patients were male. The mean BMI was 25.2 ± 3.4 kg/m², with seven patients within the normal range, five overweight, and one classified as class I obesity according to the World Health Organization (WHO) criteria [8]. Despite their relatively young age, most patients were sedentary or only occasionally active, primarily engaging in recreational football. Occupational distribution included five office workers, six manual laborers, and two unemployed individuals. Seven patients were smokers, two used chewing tobacco, and one had type 2 diabetes. All patients presented with a tendon defect ≥6 cm, measured intraoperatively with the ankle in 20° dorsiflexion. Injury mechanisms varied, including acute trauma, sometimes initially misdiagnosed as an ankle sprain, and ruptures occurring on pre-existing tendinopathy, particularly following unguided corticosteroid injections. Clinical examination consistently demonstrated a positive Thompson test, a positive Matles test, an inability to perform a single-leg heel rise, and a palpable tendon defect. Standard ankle radiographs were obtained in all cases. Additional imaging included ultrasound in six patients and MRI in two, while five patients did not undergo further imaging. Additional imaging was not systematically performed when clinical findings were considered sufficiently diagnostic. In two cases, ultrasound suggested a partial rupture, whereas complete rupture was confirmed intraoperatively, likely due to visualization of an intact plantaris tendon mimicking Achilles tendon continuity.

Outcome measures

Functional outcomes were assessed at the final follow-up using the Achilles Tendon Total Rupture Score (ATRS) [9] and the American Orthopaedic Foot & Ankle Society (AOFAS) ankle-hindfoot score [10]. Functional performance was additionally evaluated using the single-leg heel rise test. Return to physical activity and postoperative complications, including rerupture, infection, and wound healing disorders, were also recorded.

Data analysis

Given the limited sample size, only descriptive statistical analysis was performed. Continuous variables were expressed as mean ± standard deviation, and categorical variables as absolute values.

Surgical technique

**Figure 1:**
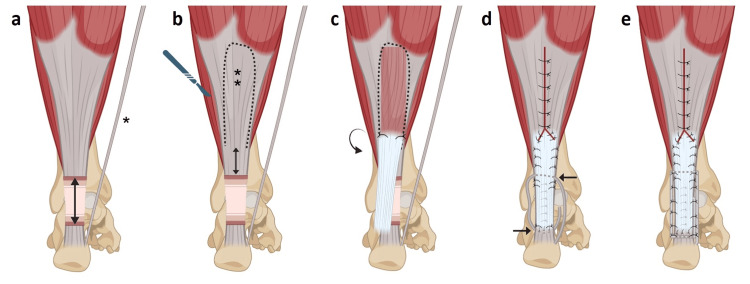
Schematic representation of the modified Rush technique with gastrosoleus fascial flap tubularization and plantaris tendon augmentation. (a) Tendon defect (↕); detached plantaris tendon (*). (b) Design of the gastrosoleus fascial flap, with a length equal to the defect length + 5 cm (**), extending distally to 2 cm above the proximal Achilles tendon stump (↕). (c) The flap is turned 180° without torsion. Two anchoring sutures secure its distal end. (d) The flap is tubularized by approximating its lateral edges along its deep surface and fixed to the distal stump. The donor site is closed. The free end of the plantaris tendon is passed through the substance of the proximal Achilles stump from medial to lateral, then through the distal stump from lateral to medial. (e) Final construct showing the fascial flap in place with plantaris tendon augmentation in a framing configuration. Image credit: This illustration was created by Dr Sifi Besma using BioRender software and reviewed by the authors for anatomical accuracy.

**Figure 2:**
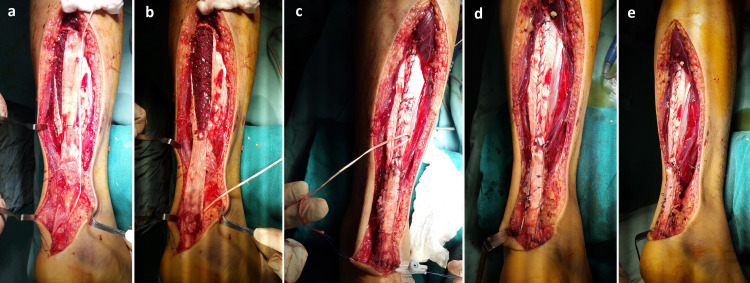
Intraoperative views illustrating the key steps of the modified Rush technique. (a) Tendon defect > 6 cm. Elevation of the gastrosoleus fascial flap, extending distally to 2 cm above the proximal Achilles tendon stump (flap length = defect length + 5 cm). The plantaris tendon is detached. (b) The flap is turned 180° without torsion. (c) The flap is tubularized by approximating its lateral edges along its deep surface and fixed to the distal stump. The donor site is closed. The free end of the plantaris tendon is whip-stitched and passed through the proximal stump from medial to lateral, then through the distal stump from lateral to medial. (d, e) Final construct showing the fascial flap in place with plantaris tendon augmentation in a framing configuration.

Reconstruction was performed using a modified Rush gastrosoleus fascial turndown flap reinforced with plantaris tendon augmentation in a framing configuration. In this series, all patients presented with an identifiable plantaris tendon. However, the surgeons were prepared intraoperatively to adapt the procedure in cases where the plantaris tendon was absent, either by relying solely on the gastrosoleus fascial flap or, depending on intraoperative findings, by converting to an alternative technique such as flexor hallucis longus tendon transfer. The patient was placed in the prone position with the feet extending beyond the table. The ankle was positioned in approximately 20° plantar flexion and the knee in 30° flexion to relax the gastrocnemius-soleus complex and facilitate tension adjustment. A pneumatic tourniquet was applied to the proximal thigh. A medial para-Achillean approach was used, minimizing subcutaneous dissection while preserving the sural nerve and small saphenous vein. Scar tissue and degenerated tendon ends were excised, and the tendon defect was measured with the ankle in 20° dorsiflexion to avoid underestimation of the gap and to reflect the physiological tension of the gastrocnemius-soleus complex. The mean tendon defect was 6.7 ± 0.8 cm. A gastrosoleus fascial flap was harvested using an inverted U-shaped design (Figures 1a-1c, 2a). The flap was approximately 2 cm wide and exceeded the defect length by 5 cm. It remained distally attached approximately 2 cm proximal to the Achilles tendon stump (Figure 1b). Two anchoring sutures were placed at the distal turning points to secure the construct and limit secondary elongation (Figure 1c). The flap was turned 180° without torsion to preserve vascularity [11], and residual muscle fibers were removed (Figures 1c, 2b). The donor site was closed using absorbable sutures (Vicryl® 2-0), allowing adjustment of flap length and tension with the ankle maintained in slight plantar flexion. The flap was then tubularized by approximating its lateral edges along the deep surface and secured to the distal stump, creating a continuous tendon bridge (Figures 1d, 2c). The plantaris tendon was sectioned proximally while preserving its calcaneal insertion and used as longitudinal reinforcement. Using a clamp and catheter as a guide, it was passed through the healthy portions of the proximal stump (medial to lateral) and distal stump (lateral to medial) until fully incorporated into the construct (Figures 1d, 2c). It was then secured to both the fascial flap and tendon stumps with interrupted absorbable sutures (Figures 1e, 2d, 2e). This combined construct provided a tubularized fascial bridge reinforced by longitudinal biological augmentation. Final tension was adjusted to reproduce physiological equinus relative to the contralateral side. Postoperatively, a cast boot was applied with the ankle in approximately 20° equinus for six weeks without weight-bearing. This was followed by a removable orthosis for an additional six weeks, allowing gradual passive rehabilitation with progressive reduction of equinus. Active rehabilitation began at three months, including progressive strengthening of the triceps surae.

## Results

At the postoperative follow-up, the mean duration was 16.1 ± 2.7 months, with all patients completing the minimum follow-up period. The mean ATRS was 81.0 ± 3.9, and the mean AOFAS score was 83.9 ± 2.3. At the final follow-up, patients performed a mean of 14.5 ± 1.3 single-leg heel raises (Table 1). One patient resumed recreational running at 10 months postoperatively. No reruptures or postoperative complications were observed. Specifically, no cases of infection, wound healing disorders, or skin complications were reported. These findings were observed despite the presence of risk factors such as smoking and diabetes in several patients. The mean BMI was 25.2 ± 3.4 kg/m². When analyzed descriptively, overweight patients had slightly lower ATRS values compared to those with normal BMI. No statistical analysis was performed due to the limited sample size, and no association between BMI and functional outcomes was established.

**Table 1:** Patient characteristics, surgical data, and postoperative outcomes. BMI = body mass index; CS = corticosteroid; ATRS = Achilles Tendon Total Rupture Score; AOFAS = American Orthopaedic Foot & Ankle Society Score

Patient	Age (years)	Sex	BMI (kg/m²)	Comorbidities	Smoking status	CS injection	Injury mechanism	Gap (cm)	Follow-up (months)	ATRS (/100)	AOFAS (/100)	Single-leg heel raises (n)
1	64	Male	25.9	None	Yes	No	Traumatic	7	12	84	86	14
2	36	Male	26.5	None	Yes	Yes	Degenerative	6	17	81	83	14
3	39	Male	23.4	None	Yes	No	Traumatic	6	14	84	86	15
4	42	Male	22.1	None	Chewing tobacco	Yes	Degenerative	6	18	85	87	16
5	33	Male	21.8	None	Yes	No	Traumatic	7	14	85	86	14
6	50	Male	31.4	Type 2 diabetes	Yes	Yes	Degenerative	7	20	72	80	13
7	45	Male	29.2	None	Yes	No	Traumatic	8	15	75	80	14
8	68	Male	24.2	None	No	No	Traumatic	7	16	82	83	15
9	43	Male	27.8	None	No	No	Traumatic	6	20	80	83	16
10	33	Male	22.9	None	Yes	No	Traumatic	6	18	81	85	15
11	40	Male	23.7	None	No	No	Traumatic	7	12	83	83	14
12	51	Male	28.6	None	No	No	Degenerative	8	15	78	83	12
13	37	Male	19.8	None	Chewing tobacco	No	Traumatic	6	18	83	86	17
Mean	44.6	—	25.2 ± 3.4	—	—	—	—	6.7 ± 0.8	16.1 ± 2.7	81.0 ± 3.9	83.9 ± 2.3	14.5 ± 1.3

## Discussion

Management of chronic Achilles tendon rupture depends primarily on defect size, tissue quality, and delay to diagnosis. Beyond several weeks, tendon retraction and scar interposition make end-to-end repair difficult or impossible [1,12]. Reconstruction must restore continuity while ensuring adequate mechanical strength and biological integration. Available techniques include V-Y advancement, tendon transfers, free grafts, and synthetic materials. Each has limitations. V-Y advancement may reduce triceps surae strength, particularly in large defects [13]. Tendon transfers, especially FHL, remain the most widely used option for large defects and provide dynamic muscular reinforcement. However, their functional cost is not negligible. Wegrzyn et al. reported a mean AOFAS score of 85.3 at 28 months following modified FHL transfer [14], while Fischer et al. demonstrated comparable functional outcomes between FHL transfer and plantaris augmentation alone (AOFAS 84 vs. 80, respectively), with the FHL group carrying a higher rate of donor-site morbidity [15]. These data suggest that the functional gain attributed to the dynamic component of FHL transfer may not systematically outweigh its morbidity, particularly in patients where preserving hallux flexor strength is functionally relevant. In this context, the functional outcomes observed in our series (ATRS = 81.0 ± 3.9, AOFAS = 83.9 ± 2.3) without any tendon sacrifice appear clinically meaningful. Free grafts and synthetic materials, for their part, carry risks of infection and inflammatory reactions [16]. Several techniques combining different biological components have been described in the literature. The association of a fascial flap with a tendon transfer may provide dynamic reinforcement but at the cost of increased morbidity and surgical complexity, without consistent evidence of superior functional outcomes [17,18]. In contrast, the use of a fascial flap alone may be insufficient in cases of large defects. The addition of the plantaris tendon, therefore, represents an interesting compromise, enhancing construct strength without increasing morbidity. Local tissue techniques are, therefore, particularly relevant, preserving functional structures and avoiding distant donor-site morbidity. The Rush technique uses a tubularized gastrosoleus fascial flap to bridge large defects, offering a tendon-like structure with favorable load distribution [4]. In our series, this technique was combined with plantaris tendon reinforcement. The mean tendon defect was 6.7 ± 0.8 cm, reflecting large and technically demanding chronic ruptures. Functional outcomes (ATRS = 81.0 ± 3.9, AOFAS = 83.9 ± 2.3) were comparable to those reported for FHL transfers, while preserving native functional tendons [14,15]. The relatively low standard deviations suggest consistent results across patients, supporting the reproducibility of the technique despite the limited sample size. Patient-related factors such as BMI may also influence outcomes following Achilles tendon reconstruction due to increased mechanical loading on the repair construct. In our series, overweight patients demonstrated slightly lower ATRS compared to normal-weight individuals, suggesting a modest negative effect. However, this trend remains limited and should be interpreted cautiously, given the small sample size and the low number of obese patients.

Biomechanically, the proposed construct relies on two complementary components: a tubularized fascial flap acting as a tendon substitute and the plantaris tendon providing longitudinal reinforcement. This combination may theoretically enhance initial stability and reduce the risk of secondary elongation. Although no direct biomechanical testing was performed, the absence of rerupture, the relatively low standard deviations observed in functional scores (ATRS ± 3.9, AOFAS ± 2.3), and the consistency of functional outcomes provide indirect support for the reliability of this construct. The preservation of vascularity may further contribute to favorable healing conditions [11]. The plantaris tendon offers advantages of proximity, ease of harvest, and negligible functional impact, though its role remains passive. Unlike tendon transfers, this approach preserves native musculotendinous structures and avoids donor-site compromise while restoring tendon continuity using local tissues.

An original finding was its potential to create diagnostic confusion on imaging. In two patients of our series, preoperative ultrasound incorrectly concluded a partial rupture, whereas a complete rupture was confirmed intraoperatively. This misdiagnosis was attributed to the visualization of an intact plantaris tendon, whose echogenicity and fibrillar pattern can closely mimic residual Achilles tendon continuity. This pitfall is not exceptional, as the plantaris tendon is present in approximately 90% of the population and runs in close anatomical proximity to the medial border of the Achilles tendon. The clinical consequences can be significant: a diagnosis of partial rupture may lead to conservative management or delayed surgical referral, allowing further tendon retraction and scar interposition, the very factors that transform an acute rupture into a complex chronic reconstruction case. When clinical findings strongly suggest complete rupture, a discordant ultrasound result should be interpreted with caution and confirmed by MRI if necessary. Dynamic ultrasound assessment, by demonstrating independent excursion of the plantaris tendon during ankle motion, may also help differentiate the two structures. Paradoxically, the same structure responsible for diagnostic confusion became a surgical asset in our technique, harvested and used as a biological reinforcement of the reconstruction. This dual role underscores the importance of systematically identifying the plantaris tendon in the preoperative workup of Achilles tendon ruptures.

The absence of complications, despite recognized risk factors such as smoking and type 2 diabetes, may be attributed to several complementary factors. The gastrosoleus fascial flap retains its proximal vascular pedicle and is turned 180° without torsion, preserving intrinsic vascularity from the outset, unlike free grafts or synthetic materials, which rely entirely on extrinsic revascularization. The plantaris tendon, harvested with its calcaneal insertion intact, similarly benefits from local vascularization. Additionally, careful soft-tissue handling, including a medial para-Achillean approach, avoidance of excessive subcutaneous dissection, and layered wound closure, likely contributed to the absence of wound complications. While these observations remain preliminary, given the small sample size, they suggest that fully biological local tissue reconstruction may offer a favorable safety profile even in patients with comorbidities.

Several limitations of this study must be acknowledged. The small sample size and absence of a control group preclude any comparative conclusions, and the findings should be interpreted strictly within the descriptive framework of a case series. In this context, the primary aim of the present study was to describe the surgical technique and to report preliminary clinical outcomes rather than to establish comparative superiority. The mean follow-up of 16.1 months, while sufficient to capture early functional recovery, may be inadequate to detect late complications such as construct elongation or rerupture. The absence of isokinetic evaluation limits objective quantification of triceps surae strength recovery, and the lack of postoperative imaging prevents assessment of biological integration and structural integrity of the construct over time. Additionally, the inclusion of exclusively male patients, while reflecting the epidemiological profile of our population, may limit the generalizability of these results to female patients, in whom hormonal and anatomical differences may influence tendon healing. Larger prospective studies incorporating isokinetic testing and systematic imaging follow-up are required to confirm these preliminary findings. Despite these limitations, this study provides preliminary clinical evidence supporting the feasibility of a fully biological reconstruction strategy using local tissues without tendon sacrifice. This technique may represent a promising alternative to tendon transfers for chronic Achilles tendon ruptures with defects ≥6 cm.

## Conclusions

The modified Rush gastrosoleus fascial turndown technique with plantaris tendon augmentation appears to be a reliable and reproducible option for the management of chronic Achilles tendon ruptures with large defects. In this preliminary series, satisfactory functional outcomes were observed, with results comparable to those reported for tendon transfers, while avoiding donor-site morbidity. As a fully biological reconstruction using local tissues, this approach may promote favorable integration while preserving vascularity and tendon excursion. Biomechanically, the combination of a tubularized fascial bridge and plantaris reinforcement may provide adequate stability, as suggested by the absence of rerupture in this cohort. This technique may represent a valuable alternative to tendon transfers for defects ≥6 cm. Further studies with larger cohorts, isokinetic evaluation, and long-term imaging follow-up are required to confirm these findings.
